# Cardiac molecular pathways influenced by doxorubicin treatment in mice

**DOI:** 10.1038/s41598-019-38986-w

**Published:** 2019-02-21

**Authors:** Ben F. Bulten, Martina Sollini, Roberto Boni, Katrin Massri, Lioe-Fee de Geus-Oei, Hanneke W. M. van Laarhoven, Riemer H. J. A. Slart, Paola A. Erba

**Affiliations:** 1 Department of Biomedical Photonic Imaging, TechMed Centre, University of Twente, Enschede, The Netherlands; 2 Department of Nuclear Medicine, Queen Beatrix Hospital, Winterswijk, The Netherlands; 3 Department of Biomedical Sciences, Humanitas University, Milan, Italy; 4 Department of Nuclear Medicine, ASST Papa Giovanni XXIII, Bergamo, Italy; 5 ASST Ovest Milanese, Ospedale Legnano via Giovanni Paolo II, Legnano, Italy; 6 Department of Nuclear Medicine, Department of Translational Research and New Technology in Medicine, University of Pisa, Pisa, Italy; 7 Division of Nuclear Medicine, Department of Radiology, Leiden University Medical Center, Leiden, The Netherlands; 8 Department of Medical Oncology, Academic Medical Center, Amsterdam, The Netherlands; 9 Department of Nuclear Medicine and Molecular Imaging, University Medical Center Groningen, Groningen, The Netherlands

## Abstract

Doxorubicin (DOX) is a potent chemotherapeutic with distinct cardiotoxic properties. Understanding the underlying cardiotoxic mechanisms on a molecular level would enable the early detection of cardiotoxicity and implementation of prophylactic treatment. Our goal was to map the patterns of different radiopharmaceuticals as surrogate markers of specific metabolic pathways induced by chemotherapy. Therefore, cardiac distribution of ^99m^Tc-sestamibi, ^99m^Tc-Annexin V, ^99m^Tc-glucaric acid and [^18^F]FDG and cardiac expression of Bcl-2, caspase-3 and -8, TUNEL, HIF-1α, and p53 were assessed in response to DOX exposure in mice. A total of 80 mice (64 treated, 16 controls) were evaluated. All radiopharmaceuticals showed significantly increased uptake compared to controls, with peak cardiac uptake after one (^99m^Tc-Annexin V), two (^99m^Tc-sestamibi), three ([^18^F]FDG), or four (^99m^Tc-glucaric acid) cycles of DOX. Strong correlations (*p* < 0.01) were observed between ^99m^Tc-Annexin V, caspase 3 and 8, and TUNEL, and between [^18^F]FDG and HIF-1α. This suggests that the cardiac DOX response starts with apoptosis at low exposure levels, as indicated by ^99m^Tc-Annexin V and histological apoptosis markers. Late process membrane disintegration can possibly be detected by ^99m^Tc-sestamibi and ^99m^Tc-glucaric acid. [^18^F]FDG signifies an early adaptive response to DOX, which can be further exploited clinically in the near future.

## Introduction

Doxorubicin (DOX) is a glycosidic antibiotic belonging to the class of anthracyclines. It is a highly effective cytotoxic drug used against both solid and hematological cancer types^1^. The counterpart of this powerful antineoplastic effect is that DOX (and anthracyclines in general) induces apoptosis in noncancerous cells, including cardiomyocytes. This causes acute and chronic cardiotoxicity (termed anthracycline-induced cardiotoxicity, or AIC), which negatively impacts patient outcome^2^.

Among the key cardiotoxic effects of DOX are the inhibition of topoisomerase IIβ, the formation of reactive oxygen species (ROS) accompanied by depletion of endogenous antioxidants, and the subsequent triggering of the intrinsic mitochondria-dependent and extrinsic death receptor apoptotic pathway^3,4^. As a result, caspase 3 is activated and, collectively, a subsequent cascade of events typical of apoptosis occurs. The cascade can be divided into three phases: an early phase consisting of caspase activation and phosphatidylserine (PS) expression; a mid-phase consisting of DNA fragmentation, chromatin condensation, and membrane phospholipid metabolization; and a late stage characterized by membrane blebbing and cell shrinkage^5^. These mutations in cardiomyocyte homeostasis underlie the pattern of subclinical cardiotoxicity. A better understanding of the involved mechanisms offers the opportunity for multi-targeted exploration of early AIC, leading to the development of non-invasive and quantitative detection tools to optimize a patient-based approach.

Available imaging techniques such as echocardiography, multigated radionuclide angiography (MUGA), and cardiac magnetic resonance imaging (CMR) have been used to accurately and reproducibly evaluate anthracycline-induced changes. However, the anatomy-based parameters provided by these approaches only detect late-phase AIC alterations, and have low sensitivity in detecting subclinical myocardial dysfunction^1,6^. Furthermore, with the increasing number of cancer survivors, there is a growing need to change the paradigm from the current method of simply measuring reduction of myocardial function to early identification of patients at risk of irreversible cardiotoxicity, thereby optimizing the benefits of protective therapy. Accordingly, the early-phase detection of subclinical AIC has become an important field of medical investigation^7^.

**Figure 1:**
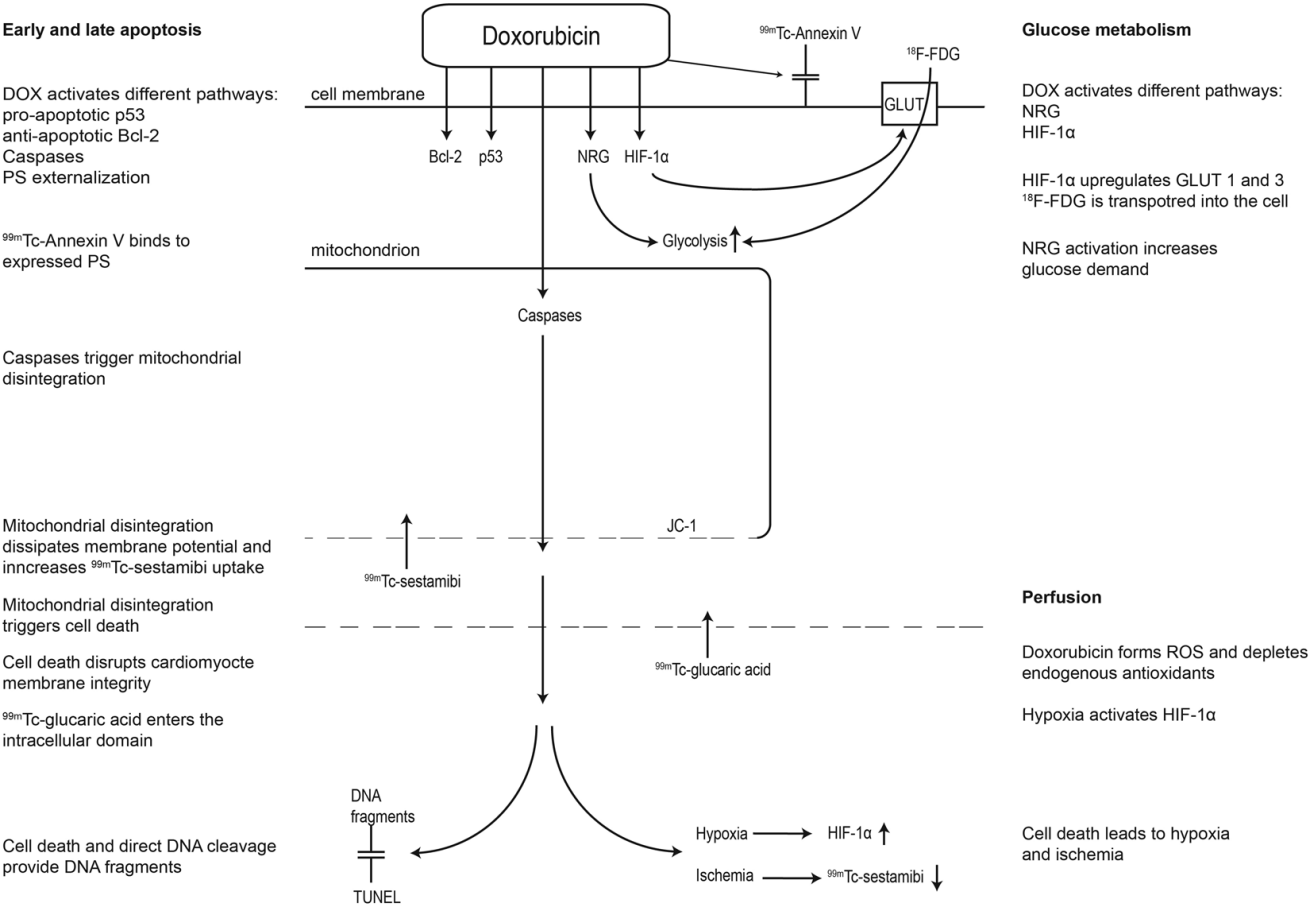
The studied cardiac markers in the process of AIC. DOX = doxorubicin, PS = phosphatyldiserine, NRG = neuregulin, GLUT = glucose transporter, ROS = reactive oxygen species.

To elucidate the mechanisms involved in the cardiotoxic process, we aimed to map the cardiomyocyte response using an animal model of DOX-induced cardiotoxicity by four different mechanistic approaches. Since early apoptosis is characterized by changes in PS expression and activation of several intracellular regulatory proteins, PS distribution was studied with ^99m^Tc-Annexin V and correlated to histological apoptosis markers caspase 3 and 8, p53, anti-apoptotic Bcl-2, and DNA fragmentation analysis. Cardiomyocyte (mitochondrial) membrane integrity (Δψm), which indicates later off-set apoptotic and necrotic changes, was assessed with ^99m^Tc-sestamibi and ^99m^Tc-glucaric acid and correlated to JC-1 fluorescent dye, Bcl-2, and DNA fragmentation. Furthermore, perfusion changes were studied using ^99m^Tc-sestamibi and correlated to hypoxia-inducible factor (HIF-) 1α, which plays an important role in the molecular response to hypoxemic conditions^8^. Finally, glucose metabolism was evaluated by 18-fluorine-fluorodeoxyglucose ([^18^F]FDG) and correlated to HIF-1α, since the latter is suggested to activate glucose transporters (GLUT)^9^. A schematic overview of the studied markers and their place in the cardiotoxic process is provided in Fig. 1.

## Materials and Methods

### Study design

**Figure 2:**
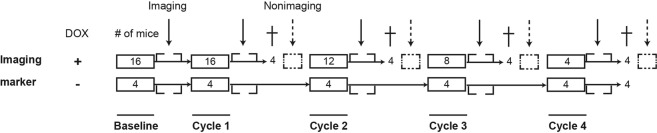
Schematic study design. Non-imaging markers are WB, IHC, and FACS expression markers. † = sacrifice of mice.

Figure 2 shows a schematic representation of the study design. Sixteen healthy male BALB/c mice were assigned to one of four radiopharmaceutical groups. Intraperitoneal (i.p.) administration of 15 mg/kg DOX was applied in all groups every three weeks for one, two, three, or four cycles, respectively. The maximum cumulative dose comprised 60 mg/kg given over 12 weeks, which corresponds to a potentially cardiotoxic dose of 1050 mg per cycle for a 70-kg male patient^10^. At baseline and after completing each cycle of DOX, the cardiac and other organ biodistribution of the four radiopharmaceuticals was investigated approximately one hour after intravenous (i.v.) tail vein administration. After a group of four mice completed the regimen, the left ventricular (LV) function was evaluated by transthoracic echocardiography. Then animals were sacrificed for non-imaging assessment, which included deoxynucleotidyl transferase biotin-dUTP nick-end labeling (TUNEL) staining, cardiac expression of Bcl-2, p53, caspase 3, caspase 8, and HIF-1α and Δψm by Western blot (WB) analysis, immunohistochemistry (IHC), and fluorescence-activated cell sorting (FACS)/flow cytometry. Furthermore, a group of four healthy BALB/c mice per radiopharmaceutical served as controls, as they were treated at the same time-points and conditions with saline administration. They were also imaged after every cycle, but were only sacrificed after completing all four saline cycles. The study was approved by the animal care committee of the University of Pisa in accordance with relevant animal care guidelines.

### Radiopharmaceutical preparation and administration

#### ^99m^Tc-sestamibi

The lipophilic cation Sestamibi (Cardiolite, Bristol-Myers Squibb) was radiolabeled by adding water to 1–3 ml of sodium pertechnetate (^99m^TcO_4_)Na and heated for ten minutes at 100 °C. After cooling, a monovalent lipophilic cationic complex was retrieved (^99m^Tc-sestamibi-6–2-metossi-isobutyl-isonitril), of which an average of 18.5 MBq in 0.1 mL was injected i.v.

#### ^99m^Tc-Annexin V

Human recombinant Annexin V (National Institutes of Health, Bethesda, MD, USA) was radiolabeled through combined *in vitro* incubation at room temperature for 30 minutes of 60–90 μg di-HYNIC-Annexin V diluted in 0.5 ml of physiological saline solution, 0.74–1.11 MBq pertechnetate anion (^99m^TcO_4_^−^), which was freshly eluted by a 6 GBq generator (Amersham, GE HealthCare), 10–20 μg of reduction agent (stannous chloride dehydrate, SnCl_2_−2H_2_O), and 1.5 mg of tricin (N-[tri(hydroxymethyl)methyl]glycine) as co-ligand. Labeling yield was 85–90%. ^99m^Tc-Annexin V was then injected in a 0.1 mL solution containing on average 18.5 MBq of ^99m^Tc-Annexin V, which corresponds to a total protein concentration of 4.5 ng per animal.

#### ^99m^Tc-glucaric acid

^99m^Tc-glucaric acid (Molecular Targeting Technologies, Inc. West Chester, PA, USA) was prepared from a lyophilized kit containing 12.5 mg of D-glucaric acid and 0.15 mg of stannous chloride^11^ by adding 1480 MBq (40 mCi) freshly eluted ^99m^Tc-pertechnetate as previously described^12^. Labeling efficiency (always >98%) was checked by ascending chromatography. The radiopharmaceutical was injected in 0.1 mL solution, containing on average 18.5 MBq ^99m^Tc-glucaric acid.

#### [^18^F]FDG

The glucose analogue [^18^F]FDG was provided as a bulk vial (Gluscan, GE Healthcare), from which a sample of 18.5 MBq in 0.1 ml was injected. To avoid the intake of carbohydrates, the mice that received [^18^F]FDG were provided a fat-enriched diet.

### Echocardiographic evaluation

LV function was evaluated with transthoracic echocardiography before the mice were euthanized, approximately 10 minutes after initiation of sedation. M-mode images were used for measurements of LV end-diastolic internal diameter (LVEDD) and LV end-systolic internal diameter (LVESD) in two consecutive beats. Fractional shortening (FS) was calculated as FS% = [(LVEDD − LVESD)/LVEDD] × 100.

### Biodistribution and histological analysis

After completion of their regimen and one hour after injection of the radiopharmaceuticals, animals were anesthetized and sacrificed. For biodistribution studies, samples of thyroid, kidney, tail, bone, lung, heart, muscle, blood, stomach, skin, liver, spleen, intestine, and urine were removed and weighed immediately. Radioactivity of these samples was registered for 180 seconds by a gamma counter (2470 Automatic Gamma Counter Wizard, PerkinElmer, Waltham, MA, USA), along with an aliquot of the injective and the tail to correct for partial extravasation, expressed as a percentage of the injected dose per gram of tissue (%ID/g).

Before gamma counting, two three-millimeter cardiac tissue slices were obtained for histology: one was frozen at −80 °C in dry ice and the other was placed in 4% formalin. After treatment with a protease inhibitor (1:100 in 10 mM Tris buffer, pH 8), apoptotic cells were assessed with direct fluorescence fragment end labeling of DNA breaks (TUNEL kit, Fluorescein FragEL, Calbiochem, San Diego, CA, USA). Caspase 3 and HIF-1α were immunostained using antihuman-mouse active antibodies (rabbit anti-caspase 3 and rabbit anti-HIF polyclonal antibody, Chemicon International, Temecula, CA, USA) after incubation with a fluorescent-labeled secondary antibody (Alexa Fluor 488 goat anti-rabbit IGg, Thermo Fisher Scientific, Waltham, MA, USA). Cells stained with TUNEL, caspase 3, and HIF-1α were counted in ten randomly selected high-power tumor fields (magnification x200) and expressed as a percentage of total cells. Furthermore, cardiac samples were analyzed by Western blotting to identify the specific amount of Bcl-2, p53 (both Santa Cruz Biotechnology Inc., Dallas, TX, USA), caspase 3, and caspase 8 (both Cell Signaling Technologies Inc., Danvers, MA, USA).

### Measurement of Δψm

Inner Δψm was measured by a single fluorescent lipophilic cation 5,5′,6,6′-tetrachloro- 1,1′,3,3′-tetraethylbenzimidazolocarbocyanine iodide (JC-1) that marks the loss of potential. For this purpose, mitochondria were isolated from myocardial tissue using a dedicated kit (Mytocondria Isolation Kit, Sigma-Aldrich, St. Louis, MO, USA) stained with JC-1 and analyzed by FACS (MACSQuant, Miltenyi Biotec, Bergisch Gladbach, Germany). JC-1, concentrated in the mitochondrial matrix of normal cells due to the electrochemical potential gradient, forms red fluorescent aggregates. Any event that dissipates the mitochondrial membrane potential prevents the accumulation of the JC-1 dye in the mitochondria. Accordingly, red stained cells represent intact mitochondria, whereas when the dye is dispersed throughout the entire cell, a shift from red (J-aggregates) to green fluorescence (JC-1 monomers) is observed. A red-to-green (i.e., intact to disrupt mitochondrial) ratio was performed and described as upper and lower border (UR and LR). Consequently, a decreasing ratio was considered a disintegration of membrane potential.

### Statistical analysis

Biodistribution and immunofluorescent data are expressed as mean and median %ID/g tissue and standard deviation (SD). Data were analyzed by non-parametrical tests (Kruskal-Wallis and Dunn) to evaluate the differences in groups at different time points and by Mann-Whitney U to test the significance between DOX-treated and DOX-naïve mice. Furthermore, Spearman’s rho was used to assess correlations. Significance was set at *p* < 0.05. Statistics were performed using IBM SPSS 20 for Windows. The datasets generated and/or analyzed as part of the current study are available from the corresponding author by reasonable request.

## Results

### Doxorubicin treatment

A total of 64 DOX-treated and 16 DOX-naïve mice were examined. The mice that received up to three cycles (45 mg/kg) of DOX did not exhibit any significant side effects and appeared healthy. The mice that received four cycles (60 mg/kg) either died or showed significant side effects such as weight loss, edema, and alopecia.

### Echocardiography

FS assessment of DOX-treated and DOX-naïve mice did not show significant differences at cycles 1 and 2 (63.3 ± 6.1% and 64.8 ± 5.3%, respectively). At cycles 3 and 4, the average FS for the DOX-treated group decreased to 48.6 ± 4.5% and 42.1 ± 3.7%, respectively, whereas the average FS for the control groups remained invariable as compared to the baseline.

### Cardiac and biodistribution of imaging biomarkers

**Figure 3:**
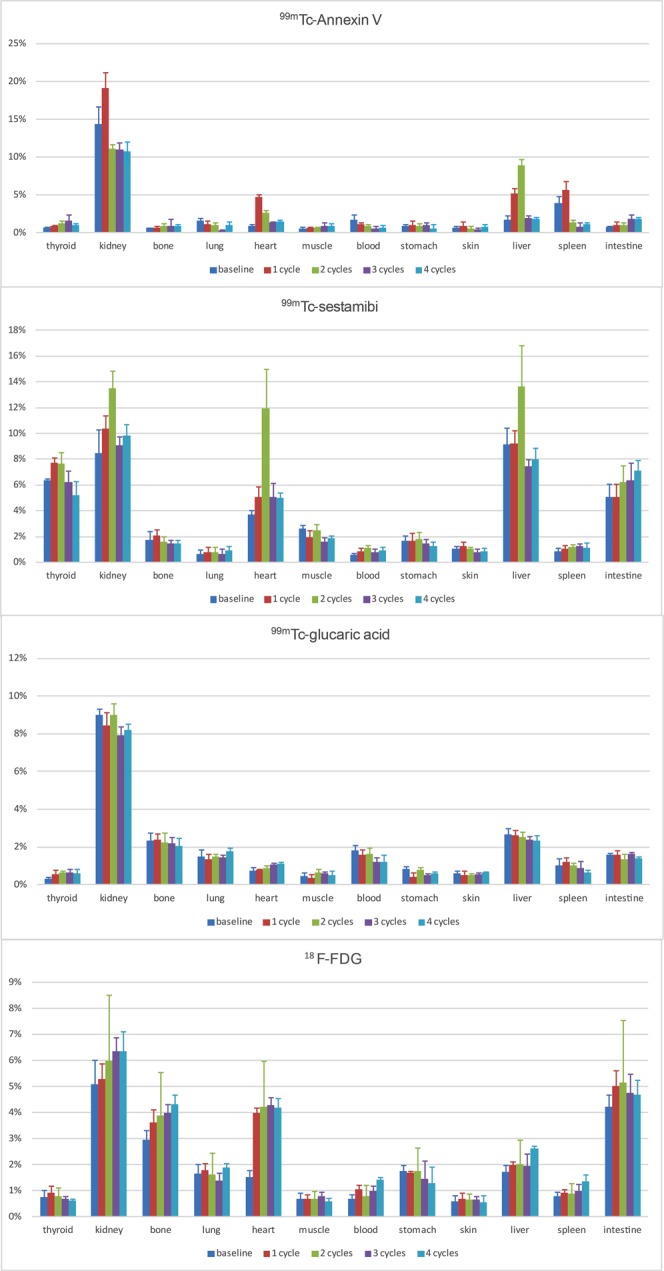
Biodistribution of radiopharmaceuticals during treatment. At baseline and after one, two, three, or four cycles of DOX administration (corresponding to a dose of 15, 20, 45, or 60 mg/kg), expressed as %ID/g.

**Figure 4:**
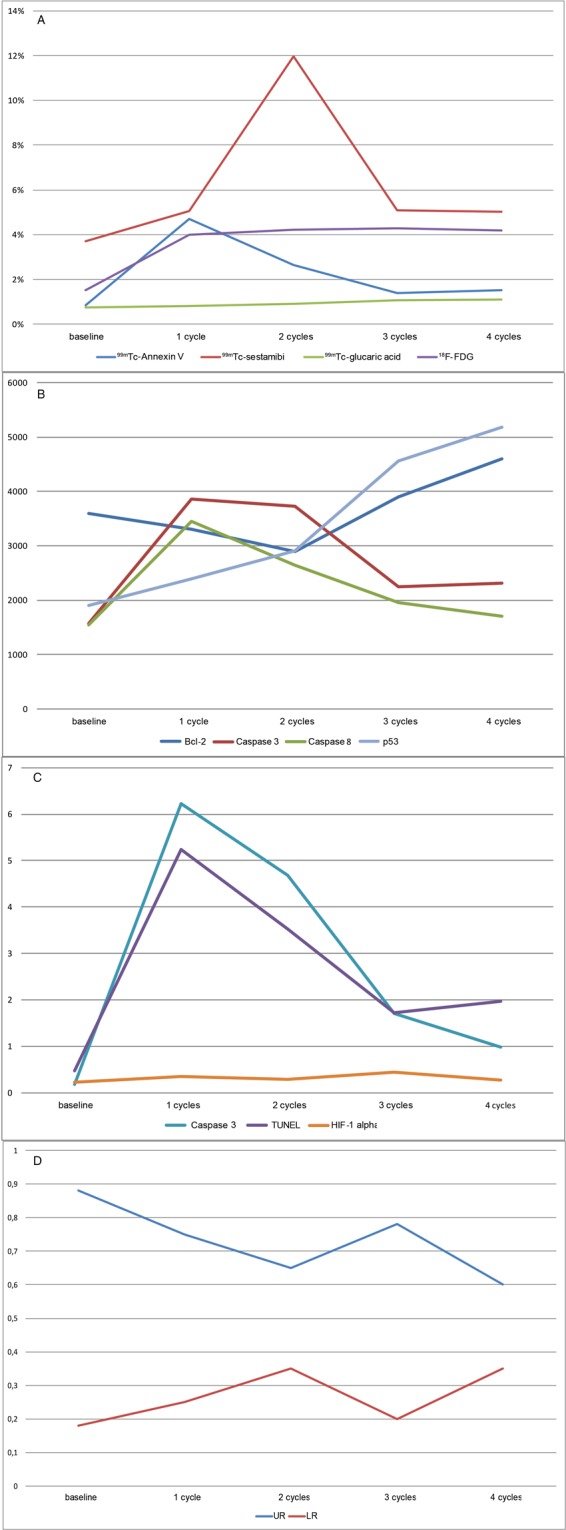
Cardiac distribution of imaging and non-imaging markers at baseline and after DOX administration. (**A**) Radiopharmaceutical uptake. (**B**) WB markers Bcl-2, caspase 3 and 8, and p53. (**C**) IHC markers caspase 3, TUNEL, and HIF-1α in percentage of positive cells. (**D**) Mitochondrial membrane potential markers. Upper and lower ratio (UR and LR).

**Figure 5:**
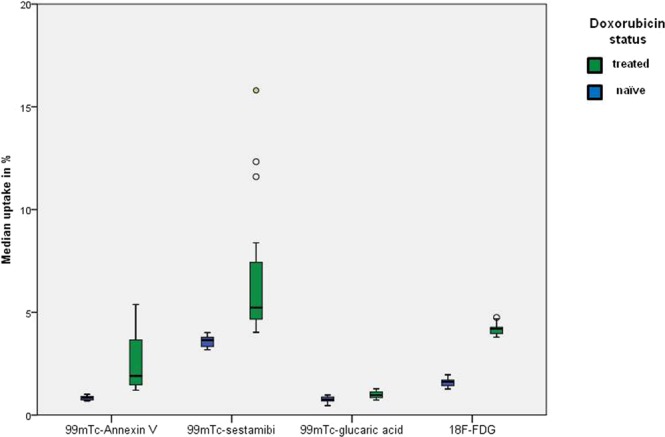
Median cardiac uptake of radiopharmaceuticals in all treatment cycles, stratified according to treatment with DOX. The median uptake differed significantly between DOX-treated and DOX-naïve mice for all radiopharmaceuticals (*p* < 0.05, Mann Whitney U).

The biodistribution of radiopharmaceuticals is shown in Figs 3–5 and summarized in Table 1.

**Table 1:** Cardiac uptake of investigated markers.

	DOX status	Median uptake in % (SD)					*p*-value^†^
		*Baseline*	*1 cycle*	*2 cycles*	*3 cycles*	*4 cycles*	
^*99m*^ *Tc-sestamibi*	Treated	3.71 (0.003)	5.06 (0.004)	**11.97** (0.030)	5.09 (0.011)	5.02 (0.004)	0.007
	Naïve		**3.88** (0.003)	3.79 (0.002)	3.45 (0.002)	3.40 (0.002)	*ns*
	Difference		1.18*	8.18*	1.64*	1.62*	
^*99m*^ *Tc-Annexin V*	Treated	0.86 (0.002)	**4.69** (0.008)	2.65 (0.003)	1.40 (0.001)	1.52 (0.002)	0.002
	Naïve		0.82 (0.001)	**0.90** (<0.001)	0.81 (0.002)	0.82 (0.001)	*ns*
	Difference		3.87*	1.75*	0.59*	0.70*	
^*99m*^ *Tc-glucaric acid*	Treated	0.75 (0.002)	0.80 (0.001)	0.90 (0.001)	1.07 (0.001)	**1.10** (0.001)	0.009
	Naïve		0.63 (0.001)	0.70 (0.001)	**0.88** (0.001)	0.87 (0.001)	*ns*
	Difference		0.17*	0.20*	0.19*	0.23*	
[^18^F]FDG	Treated	1.51 (0.003)	4.00 (0.002)	4.22 (0.002)	**4.28** (0.003)	4.18 (0.004)	0.03
	Naïve		1.48 (0.001)	1.63 (0.002)	**1.67** (0.002)	**1.67** (0.002)	*ns*
	Difference		2.52*	2.59*	2.61*	2.51*	
		**Median expression by WB (SD)**					
*Bcl-2*	Treated	3560 (773)	3305 (437)	2790 (246)	3850 (250)	**4750 (189)**	0.009
*p53*		1908 (79)	2395 (132)	2905 (358)	4565 (273)	**5185 (189)**	0.001
*Caspase 3*		1575 (234)	**3815 (220)**	3715 (208)	2255 (172)	2325 (211)	0.002
*Caspase 8*		1550 (173)	**3420 (288)**	2630 (215)	1895 (163)	1700 (335)	0.003
		**% of positive cells (median of 10 fields) by IHC (SD)**					
*TUNEL*		0.48 (0.14)	**5.42 (0.64)**	3.56 (0.31)	1.74 (0.20)	1.95 (0.24)	0.001
*HIF-1α*		0.22 (0.05)	0.36 (0.06)	0.29 (0.02)	**0.44 (0.06)**	0.27 (0.03)	0.005
*Caspase 3*		0.18 (0.04)	**6.28 (0.58)**	4.63 (0.40)	1.66 (0.64)	0.98 (0.23)	0.001

*p-value < 0.05 (Mann-Whitney U). ^†^*p*-value (Kruskal-Wallis). Bold = highest uptake in time; DOX = doxorubicin; SD = standard deviation; WB = Western blot; IHC = immunohistochemistry.

#### ^99m^Tc-sestamibi

Maximal cardiac uptake of ^99m^Tc-sestamibi was seen after two cycles of DOX, reaching a maximum dose of 12.0%, before dropping. Pairwise comparison returned an adjusted significance of *p* = 0.002 at two cycles. Other organs that displayed a peak dose at two cycles were the kidney and liver (13.5% and 13.7%, respectively). The measured dose in thyroid and intestinal tissue was considerable, peaking at one cycle (7.7%) and four cycles (7.1%), respectively.

#### ^99m^Tc-Annexin V

The maximal cardiac uptake of ^99m^Tc-Annexin V was 4.7% after one cycle of treatment (adj. *p* = 0.001), gradually decreasing to approximately baseline value at four cycles. The highest organ doses were measured in the kidney (19.2%, one cycle), spleen (5.6%, one cycle), and liver (8.9%, two cycles). In other organs only small amounts of ^99m^Tc-Annexin V were measured.

#### ^99m^Tc-glucaric acid

The kidney showed the highest uptake of ^99m^Tc-glucaric acid, particularly at baseline and two cycles (both 9.0%). Cardiac uptake was the lowest compared to the other radiopharmaceuticals and did not show a significant increase with DOX dose. It peaked at four cycles (1.1%).

#### [^18^F]FDG

[^18^F]FDG uptake was the highest in the kidney, mainly after three and four cycles (6.4% and 6.3%). Cardiac uptake rose after one week to its peak at three cycles (4.3%, adj. *p* = 0.02). Other organs that displayed high uptake were bone (marrow), liver, and intestine (peak dose at four, four, and two cycles, respectively).

### Cardiac expression of non-imaging markers

Bcl-2 expression reached its minimum at two cycles, although not significant, before increasing to the maximum at four cycles. Expression of p53 increased with dose, peaking at four cycles (adj. *p* = 0.001). Caspase 3 expression showed an identical trend by both WB and IHC measurement, peaking after the first cycle of DOX (with an adj. *p* = 0.004 and adj. *p* = 0.001, respectively), but decreasing with subsequent cycles to near baseline levels. Caspase 8 expression showed a similar trend, with a maximum at one cycle of DOX (adj. *p* = 0.007), before decreasing to baseline level. TUNEL also peaked at one cycle (adj. *p* = 0.001) and dropped with subsequent cycles, although it did not return to baseline level. HIF-1α showed an increase in percentage of positive cells and peaked at 0.45 after three cycles (adj. *p* = 0.004), then dropped to 0.27 at maximum DOX dose.

JC-1 expression, depicting Δψm, showed a decrease of UR during the first two cycles, shortly increased after three cycles, and subsequently decreased after the last cycle. The LR increased initially, but dropped after three cycles and then rose again during the last cycle.

### Correlations

**Figure 6:**
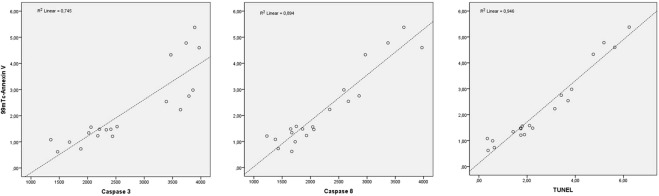
Scatter plot of ^99m^Tc-Annexin V correlations (ρ > 0.80).

Table 2 shows the calculated Spearman’s ρ used to determine correlations between imaging and non-imaging markers, stratified according to the metabolic pathway. Most striking were the correlations between ^99m^Tc-Annexin V and the caspases and TUNEL (Fig. 6). Less strong, though significant, were the relationships between ^99m^Tc-Annexin V and Bcl-2 (ρ-0.50), ^99m^Tc-sestamibi and Bcl-2 (ρ-0.55), and ^18^F-FDG and HIF-1α (ρ 0.60). JC-1 measurements were insufficient to calculate exact correlations.

**Table 2:** Correlations of investigated markers stratified to studied mechanism.

	Bcl-2	Caspase 3 (WB)	Caspase 8	p53	TUNEL	HIF-1α	Caspase 3 (IHC)
***Early apoptosis***							
^99m^Tc-Annexin V	−0.50*	0.91^†^	0.89^†^	0.08	0.95^†^		0.92^†^
***Late apoptosis***							
^99m^Tc-sestamibi	−0.55*				0.38		
^99m^Tc-glucaric acid	0.28				−0.04		
***Perfusion***							
^99m^Tc-sestamibi						0.17	
***Glucose metabolism***							
[^18^F]FDG						0.60^†^	

Correlations assessed by Spearman’s ρ. *p < 0.05. ^†^*p* < 0.01. WB = Western blot; IHC = immunohistochemistry.

## Discussion

This is the first study that evaluated the cardiac response to anthracycline exposure from several mechanistic molecular viewpoints, including early and late apoptosis imaging, perfusion changes, and glucose metabolism by a combination of molecular imaging and histological biomarkers. As previously mentioned the drop of LV ejection fraction (LVEF) represents a late-phase phenomenon in the physiopathology of chemotherapy-induced cardiotoxicity and has low sensitivity in the presence of subclinical myocardial dysfunction. Many patients present with histological evidence of anthracycline-related changes and normal resting LV function^13^. This evidence has led researchers to look to other methods for evaluating cardiac function, regardless of cardiac volume changes, to detect the earliest manifestation of cardiotoxicity. However, the pathologic mechanisms underlying cardiotoxicity remain unclear. The results of our study, which map the patterns of different radiopharmaceuticals as surrogate markers of specific metabolic pathways (i.e., glucose consumption and perfusion) induced by chemotherapeutic administration, provide a direction for future research.

### PS expression

^99m^Tc-Annexin V allows apoptosis imaging by binding to PS molecules at the outer surface of apoptotic cell membranes^7^. The exposure and redistribution of PS marks the start of the apoptotic execution phase. Elevated myocardial expression in DOX-treated animals has been associated with the presence of cardiac oxidative stress^14^. In patients treated with Adriamycin, ^99m^Tc-Annexin V imaging detected cardiomyocyte death at a lower dose and an earlier stage than echocardiography^15^.

In the current study, we observed a markedly increased expression of ^99m^Tc-Annexin V after the first cycle of DOX. This suggests that apoptosis begins at low levels of anthracycline exposure. Bennink *et al*. observed a similar early rise of ^99m^Tc-Annexin V uptake in rat hearts, also demonstrating a dose-dependent increase^16^. In contrast, we noted a decrease in ^99m^Tc-Annexin V uptake with subsequent cycles of DOX. This finding was confirmed by a strong correlation between ^99m^Tc-Annexin V and histological apoptosis markers caspase 3 and 8, which support a real reduction of apoptotic PS expression. Most likely, only a limited number of cardiomyocytes are prone to anthracycline-induced apoptosis, which already occurs at low cumulative dose. This selective induction of apoptosis in susceptible cardiomyocytes has been observed before by Bennink *et al*.^16^.

Our results indicate a strong relationship between TUNEL activity and ^99m^Tc-Annexin V expression. This was different from that reported by Bennink *et al*.^16^. However, for adequate TUNEL activity measurement, cardiac tissue must be examined extensively and, as already suggested, TUNEL activity was undetectable, likely due to an insensitive method. Indeed, recent research has indicated that DNA degradation belongs to the earliest events of the apoptosis cascade^5^.

### Membrane disintegration

Cell blebbing, shrinkage, and membrane disintegration occur during the late phase of the apoptotic cascade. ^99m^Tc-glucaric acid (glucarate), binding positively charged nuclear histones in the intracellular domain after cardiomyocyte membrane disintegration, may be used as a marker of the membrane disintegration. In contrast to the early apoptotic changes that can be reversed, cardiomyocyte membrane disintegration represents irreversible damage in necrotic cells^17^. This is illustrated by a distinct expression of ^99m^Tc-glucaric acid in necrosis, compared to the absence of ^99m^Tc-glucaric acid signal in apoptotic cells, as demonstrated by Khaw *et al*.^18^. We observed a discrete, though significant, elevation of ^99m^Tc-glucaric acid compared to controls, which minimally but consistently increased with subsequent doses. Since irreversible cell damage is known to be dose-dependent, the observed trend of ^99m^Tc-glucaric acid suggests that it actually depicts irreversible cardiomyocyte damage as shown by Okada *et al*.^17^, a finding of high potential in AIC. However, the differences were discrete and stratification into high- and low-risk groups by imaging method could therefore be problematic. Furthermore, we observed a (non-significant) increasing trend among control mice between baseline and cycle four. This could mean that ^99m^Tc-glucaric acid also depicts physiologic cell turnover. Additionally, fructose transporter GLUT-5 is suggested to mediate ^99m^Tc-glucaric acid uptake in viable cells. This transporter is also present on murine cardiomyocytes; therefore, GLUT-5 upregulation in response to doxorubicin exposure may also play a role as well^19,20^. The extent of these effects is unknown and the interpretation of fluctuations in ^99m^Tc-glucaric acid uptake in particular is thus challenging and warrants additional research.

In addition to cardiomyocyte membrane disintegration, mitochondrial membrane integrity is compromised in AIC. ^99m^Tc-sestamibi, positively charged, is taken up in normal functioning cardiomyocytes due to a large negative mitochondrial transmembrane potential. It has been shown *in vitro* that, in response to clinically relevant doxorubicin levels, opening of calcium-regulated transition pores in the mitochondrial membrane dissipates membrane potential^21^. Additionally, it has been suggested that monoclonal antibodies (e.g., trastuzumab) partly exert their cardiotoxic effect by reducing membrane potential^22^. In doxorubicin-treated patients, increased ^99m^Tc-sestamibi uptake and retention in myocardial mitochondria has been observed^23^. In our study, DOX-treated animals showed a significant increase in cardiac uptake of ^99m^Tc-sestamibi in two cycles, followed by a decrease during cycles three and four. This could be explained by the fact that mitochondrial membrane disintegration peaks at medium anthracycline exposure, a finding supported by the observation that Δψm dropped at two cycles. However, ^99m^Tc-sestamibi kinetic is complex and influenced by some factors such as p-glycoprotein (p-gp) expression. P-gp is a membrane efflux transporter that pumps toxic substances, including DOX, out of cells. ^99m^Tc-sestamibi is a known substrate for this protein^24^. When p-gp is upregulated in response to DOX exposure, as observed in murine cardiomyocyctes^25^, ^99m^Tc-sestamibi uptake decreases accordingly. Unfortunately, we did not evaluate p-gp expression in our model and, to our knowledge, the relationship between p-gp and ^99m^Tc-sestamibi in cardiomyocytes has not been investigated before.

### Perfusion changes

^99m^Tc-sestamibi uptake is also influenced by vascular changes that occur following anthracycline administration, which resemble the effects of ischemia, illustrated by both reversible and fixed defects^23^. The observed decrease in ^99m^Tc-sestamibi could therefore also reflect blood perfusion changes dominating the above-mentioned mechanisms in case of high cumulative DOX doses. To clarify this point, it could be helpful to evaluate the uptake patterns of other ischemia tracers such as 82-Rubidium, which are incorporated into cardiomyocytes by a different transporter than ^99m^Tc-sestamibi^26^.

### Glucose metabolism

In the current study, we observed a progressive increase in [^18^F]FDG directly after commencing anthracycline treatment, which remained stable during subsequent doses of DOX. Enhanced [^18^F]FDG uptake in anthracycline-exposed cardiomyocytes may rely on a neuregulin (NRG)-mediated response. NRG is a protein secreted by the endocardium and endothelium of cardiac vasculature that might protect against AIC and, as an adaptive pattern, enhances glucose metabolism of the heart^27^. However, since the magnitude of [^18^F]FDG changes over time is of borderline significance and the potential NRG effect on [^18^F]FDG has not yet been explored, further research in this field is needed. Based on the hypothesis that patients with higher baseline NRG expression exert a better adaptive response to anthracyclines, low cardiac [^18^F]FDG uptake after anthracycline therapy could be used to identify individuals at high risk of developing AIC during the early phase of the process^28^. Such variable [^18^F]FDG uptake patterns have been observed in anthracycline-treated lymphoma patients^29^. Additionally, [^18^F]FDG can be used to assess cardiomyocyte viability, since intact cardiomyocytes utilize glucose for cell functions. Consequently, cardiomyocyte decay is accompanied by decreased glucose uptake. However, it should be noted that [^18^F]FDG application in this clinical setting requires adequate patient preparation to reduce the physiological uptake of [18F]FDG of the myocardium. The increased cardiac uptake of [^18^F]FDG throughout the cumulative anthracycline administration in our study suggests a reversible mechanism, since apoptosis apparently does not induce cell death. This is also underlined by the discrete increase of ^99m^Tc-glucaric acid, which indicates only a limited amount of irreversible cell disintegration.

We observed a strong correlation between [^18^F]FDG and HIF-1α—the latter being a hypoxia-driven transcription factor that regulates the expression of a variety of genes in response to a lack of oxygen^8^. Recently, Seino *et al*. reported a similar relationship in benign abdominal tumors^9^. They suggested that HIF-1α expression is enhanced directly after exposure to hypoxia and subsequently activates GLUT-1 and GLUT-3, hexokinase, vascular endothelial growth factor, and several glycolytic enzymes. Furthermore, since maximal [^18^F]FDG uptake and HIF-1α expression were located in areas of increased cellularity, but decreased vascularity, [^18^F]FDG accumulation is not influenced by increased vascular availability or endothelial dysfunction of the radiopharmaceutical, but results from promoting glucose uptake mechanisms as an adaptive mechanism.

Our findings indicate that [^18^F]FDG can be used not only to stratify patients into low- and high-risk groups, but also to detect reversibility without being influenced by vascular availability. This is of major importance for guiding protective therapy. It provides an interesting opportunity for clinical use, since [^18^F]FDG is now widely available and used extensively. Another major advantage of [^18^F]FDG above other imaging markers is that the accumulation can be assessed quantitatively using standard uptake value (SUV), which provides an independent inter-patient and follow-up method.

## Conclusion

In conclusion, this study demonstrated that the cardiac response to anthracyclines at low doses starts with apoptosis depicted by ^99m^Tc-Annexin V and confirmed by histological apoptosis markers. Late apoptotic and necrotic changes, such as membrane disintegration, can potentially be detected by ^99m^Tc-sestamibi and ^99m^Tc-glucaric acid. However, ^99m^Tc-sestamibi uptake is influenced by other mechanisms, including p-gp expression and vascular changes, which complicates interpretation of observed uptake patterns. ^99m^Tc-glucaric acid uptake differed slightly from controls, which implies a problematic differentiation between high and low cardiac toxicity risk. [^18^F]FDG uptake signifies an early adaptive response to DOX and has the highest potential to serve as a clinically exploitable early marker of reversible cardiotoxicity in the near future.

## Data Availability

Data for reproduction is available through the corresponding author.
